# GARP as an Immune Regulatory Molecule in the Tumor Microenvironment of Glioblastoma Multiforme

**DOI:** 10.3390/ijms20153676

**Published:** 2019-07-26

**Authors:** Niklas Zimmer, Ella Kim, Jonathan Schupp, Bettina Sprang, Petra Leukel, Fatemeh Khafaji, Florian Ringel, Clemens Sommer, Jochen Tuettenberg, Andrea Tuettenberg

**Affiliations:** 1 Department of Dermatology, University Medical Center Mainz, 55131Mainz, Germany; 2 Department of Neurosurgery, University Medical Center Mainz, 55131 Mainz, Germany; 3 Translational Neurooncology Research Group, Johannes Gutenberg University, 55131 Mainz, Germany; 4 Institute of Neuropathology, University Medical Center Mainz, 55131 Mainz, Germany; 5 Department of Neurosurgery, SHG-Klinikum Idar-Oberstein, 55743 Idar-Oberstein, Germany

**Keywords:** glioblastoma, GARP, tumor microenvironment, immunotherapy, regulatory T cells

## Abstract

Glycoprotein A repetition predominant (GARP), a specific surface molecule of activated regulatory T cells, has been demonstrated to significantly contribute to tolerance in humans by induction of peripheral Treg and regulatory M2-macrophages and by inhibition of (tumorantigen-specific) T effector cells. Previous work identified GARP on Treg, and also GARP on the surface of several malignant tumors, as well as in a soluble form being shedded from their surface, contributing to tumor immune escape. Preliminary results also showed GARP expression on brain metastases of malignant melanoma. On the basis of these findings, we investigated whether GARP is also expressed on primary brain tumors. We showed GARP expression on glioblastoma (GB) cell lines and primary GB tissue, as well as on low-grade glioma, suggesting an important influence on the tumor micromilieu and the regulation of immune responses also in primary cerebral tumors. This was supported by the finding that GB cells led to a reduced, in part GARP-dependent effector T cell function (reduced proliferation and reduced cytokine secretion) in coculture experiments. Interestingly, GARP was localized not only on the cell surface but also in the cytoplasmatic, as well as nuclear compartments in tumor cells. Our findings reveal that GARP, as an immunoregulatory molecule, is located on, as well as in, tumor cells of GB and low-grade glioma, inhibiting effector T cell function, and thus contributing to the immunosuppressive tumor microenvironment of primary brain tumors. As GARP is expressed on activated Treg, as well as on brain tumors, it may be an interesting target for new immunotherapeutic approaches using antibody-based strategies as this indication.

## 1. Introduction

Glioblastomas (GBs) are characterized by a particularly aggressive behavior, including infiltrating tumor cells in the surrounding brain tissue. After surgical removal of the primary tumor, recurrence derived from these tumor cells nearly always occurs. In addition, tumor cells are able to suppress immune responses through regulatory cells such as microglial cells or invading regulatory T cells, especially in the relapse situation [1,2,3].

Immunotherapies are considered a promising method for treatment of cancer patients in general. In this study, the patient’s own immune cells are conditioned to recognize and combat structures of the tumor. The systemic effect of the immune cells also allows scattered tumor cells to be reached. However, very little is known about the factors influencing the immigration of immune cells, as well as an effective immune response, in the tumor milieu of primary brain tumors such as GB, both in the primary tumor tissue and in the recurrent tissue. Furthermore, in many tumor entities, active suppression of the immunological defense in tumor patients significantly limits the success, in particular of immunotherapies [4]. In addition to general immunological tolerance mechanisms through regulatory T cells or tolerogenic dendritic cells (tolDC), the tumor itself also develops immune escape mechanisms. Thus, by generating an inhibitory micromilieu, efficient antitumor responses are switched off or prevented, limiting the effectiveness of immunotherapeutic approaches. 

Therefore, many studies aim to characterize new regulatory molecules and signaling pathways of tumor cells and their impact on the tolerogenic properties of the tumor microenvironment in order to identify new targets for immunotherapeutic approaches.

Despite extensive experimental and clinical research, GB is still one of the most fatal tumors in humans with a median progression-free interval with maximal therapy of less than 12 months and a median overall survival of up to 15 months [5].

In addition to the standard procedures of surgery and radiation, as well as concomitant chemotherapy, immunotherapy appears to be a promising therapeutic approach, for example, by vaccination [6]. It is known that GB, similar to other malignant tumors of the neuroectoderm (e.g., malignant melanoma has some very immunogenic surface molecules. Already, there is some research with different vaccination strategies [6,7,8] providing partly contradictory results. However, vaccination strategies have not yet been standardized or optimized, and successful phase III studies have thus far been lacking [9]. A key mechanism that usually precludes successful tumor immunotherapy is the active suppression of immunological defense [10,11,12]. In this context, general immunological tolerance mechanisms play an important role, for example, regulatory cells of the immune system, such as regulatory T cells (Treg) and microglia but also soluble factors such as interleukin-10 (IL-10) and transforming growth factor beta (TGF-β), induce an immunosuppressive environment and promote tumor progression, infiltrative growth/migration, and tumor recurrence [13,14].

The tumor itself also develops numerous so-called immune-escape mechanisms that help to shut down or prevent an efficient antitumor response. This mainly includes the genetic instability of tumor cells, which leads to changes in the surface profile or the antigenic structures on the tumor cell itself and downregulation of human leukocyte antigens(HLA) molecules [15]. Furthermore, soluble factors such as IL-10 and TGF-β, as well as pro-angiogenic factors (vascular endothelial growth factor -VEGF, platelet-derived growth factor - PDGF, fibroblast growth factor -FGF, IL-8), play a role in turning off the effector cells present in the tumor and promoting tumor angiogenesis [16,17].

We have recently shown that the specific Treg activation marker GARP (glycoprotein A repetition predominant) in its soluble form has tolerance-inducing functions [18]. GARP is a transmembrane protein whose extracellular portion consists of 21 leucine-rich domains and is expressed on both Treg and platelets [19,20]. GARP is required for the formation and surface expression of latent TGF-β [19]. In addition to its expression on activated Treg, we have also shown its occurrence on cells of primary malignant melanoma and melanoma cerebral metastasis.

In the present study, we analyzed GARP as a potential marker molecule and key factor for the immunoregulatory environment in GB and investigated its relevance as a potential target for a therapeutic approach in patients with cerebral cancer.

## 2. Results

### 2.1. GARP Expression on Immunohistochemistry of Glioblastoma and Low-Grade Astrocytomas 

Recent studies of our own group revealed GARP as an immunoregulatory molecule expressed on activated Treg and capable of suppressing effector cell proliferation and cytokine production and to confer suppressive activity to T effector cells. In addition, GARP has been detected on melanoma cells, as well as on brain metastasis of melanoma [21].

**Figure 1:**
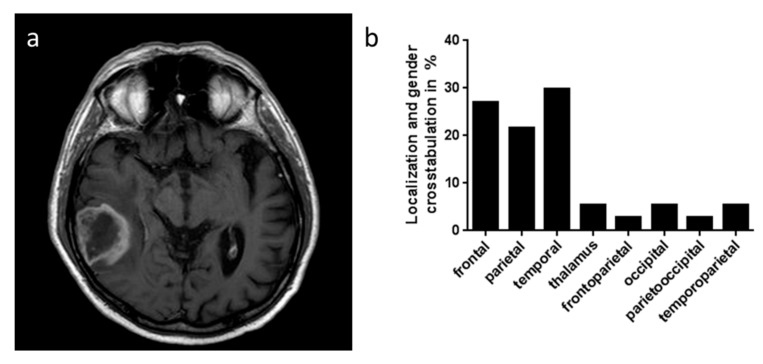
(**a**) T1-weighted gandolinium enhanced cranial axial image with a typical glioblastoma (GB) in the right dorsal temporal lobe. (**b**) Frequency of localization of GB in % is shown. Most tumors were found in the frontal, parietal, and temporal lobe of the patients.

In order to investigate in situ GARP expression and thus its relevance on the immunosuppressive tumor microenvironment of GB, 37 patients (26 males and 11 females) with histologically proven GB between January 2009 and May 2015 were included (Table 1). The mean ± standard deviation (SD) at the onset of disease for males was 69.05 ± 11.93 years and 71.38 ± 11.72 years for females (independent *t*-test *p* > 0.05). As shown in Table 1, 67.6% (17 males and eight females) had the tumor left hemispheric. The temporal lobe was the most involved part of the tumor (29.7%, Pearson Chi-square *p* > 0.05, Figure 1a). There were 33 subjects who underwent a surgical resection. The first histological diagnosis showed GB in 89.2% of the subjects. After surgery, 29 patients had radiation therapy, 26 had chemotherapy, and a combined chemoradiotherapy had been applied to 24 patients. The mean survival after diagnosis was 11.07 ± 13.27 months. 

**Figure 2:**
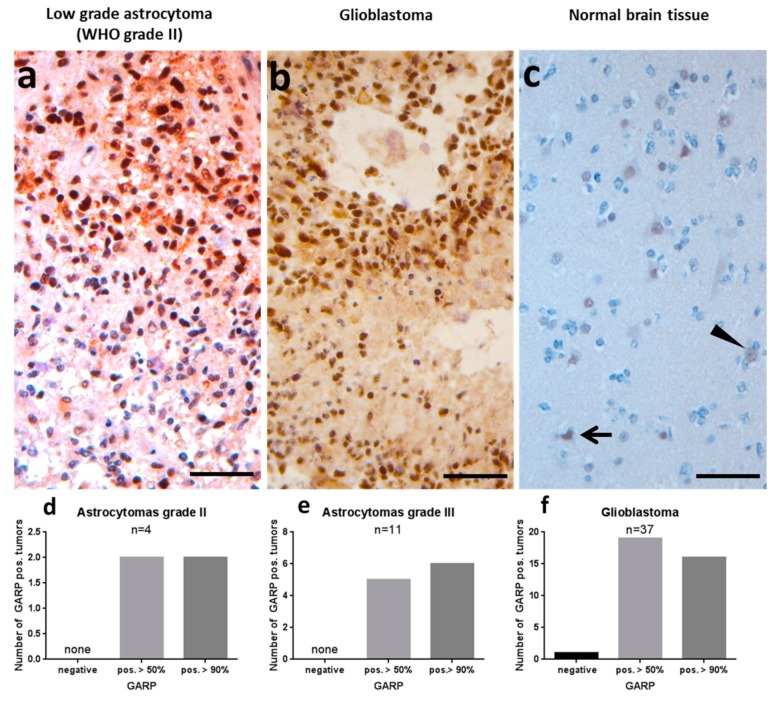
Glycoprotein A repetition predominant (GARP) immunohistochemistry in gliomas and astrocytomas. (**a**,**d**,**e**) Low-grade astrocytoma (WHO grade II) with more than 50% positive (pos.) labeled nuclei (magnification × 400). (**b**,**f**) GB with palisading necroses and more than 90% pos. stained tumor cells (magnification × 400). (**c**) Largely normal brain tissue in the neighborhood of a glioma with some labeled neurons (arrow) while others where unstained (arrowhead). Bar corresponds to 50 µm.

In these patients we investigated the relevance of GARP in primary brain tumors such as GB and compared it to astrocytomas grade II and grade III (Supplementary Tables S1 and S2). In this study, GARP immunostaining was analyzed only in tumor cells and not in inflammatory cells. Interestingly, all tumors analyzed, except one GB, showed at least 50% GARP expression (Figure 2). In detail, two of the grade II astrocytomas showed more than 50% labeled nuclei, the other two more than 90% labeled nuclei (Figure 2a,d). Five of the grade III astrocytomas showed more than 50% labeled nuclei, the other six more than 90% labeled nuclei (Figure 2e). One of the GBs was completely negative, whereas, 19 GBs showed more than 50% labeled nuclei, the remaining 16 showed more than 90% labeled nuclei (Figure 2b,f). As a control, normal brain tissue derived from the neighborhood of a glioma was stained. Single neurons, so-called dark neurons or hypoxic-ischemic damaged neurons, displayed some weak GARP staining (Figure 2c), whereas, the majority of cells did not display any GARP expression. 

Taken together these data show dominant expression of the inhibitory GARP molecule also in primary brain tumors such as GB and low-grade glioma, implicating a potential relevance for the immunosuppressive tumor micromilieu.

### 2.2. GARP Expressed on the Surface of GB and in the Cytoplasma and Nucleus

GARP is a transmembrane protein that presents latent TGF-β1 on the surface of Treg. TGF-β1 influences a variety of immune cells by conferring immune tolerance and has been shown to be present in brain tumors being associated with poor prognosis of patients with GB [22].

**Figure 3:**
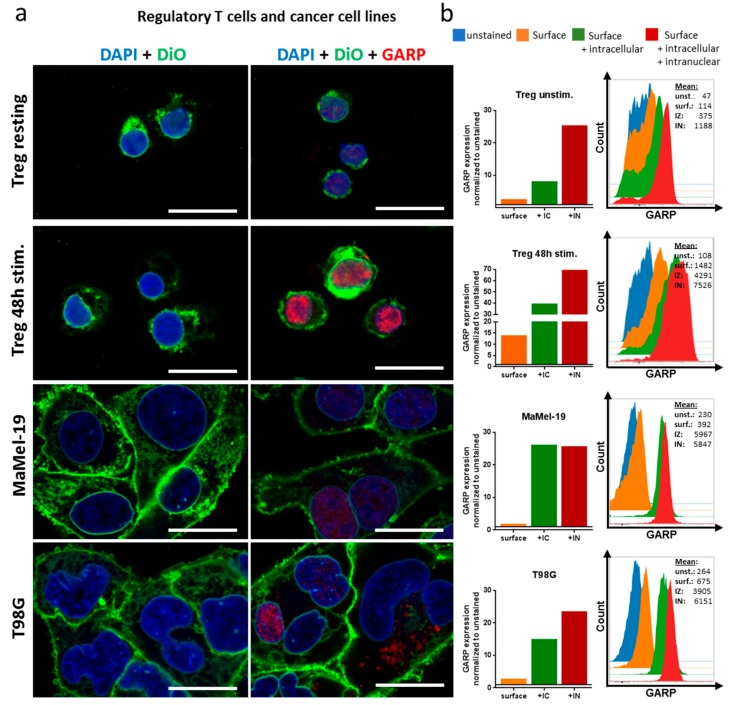
Analysis of the GARP localization in resting and stimulated regulatory T cells, melanoma cell line MaMel-19, and glioblastoma cell line T98G. The Treg were stimulated with 1 µg/mL anti-CD3 mAb, 1 µg/mL anti-CD28 mAb, and 10 IU/mL IL-2 for 48 h. (**a**) Cytoplasmatic and intranuclear localization of GARP shown in confocal images. The white bar corresponds to 20 µm (**b**) flow cytometric analysis GARP expression on the surface; surface and intracellular (IC); and surface, IC, and intranuclear (IN) of Treg, melanoma, and GB cell lines. Means were normalized to the unstained control.

**Figure 4:**
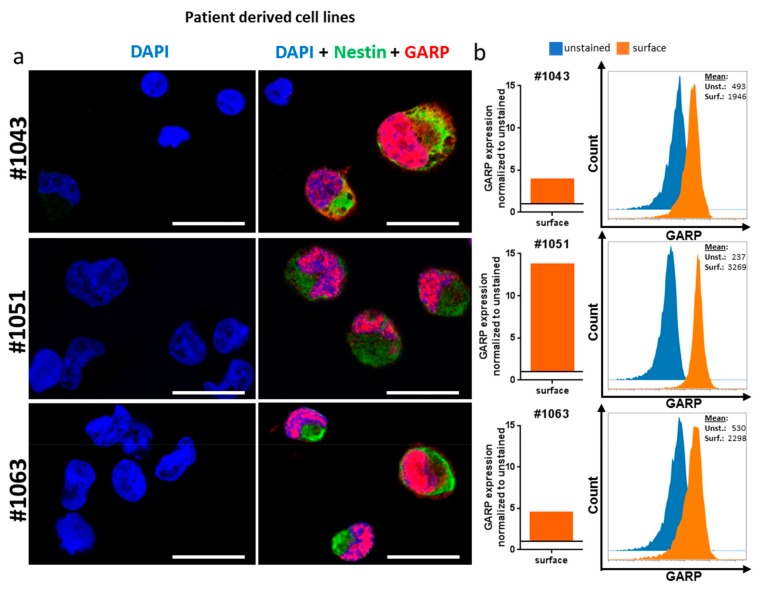
Flow cytometric and confocal analysis of GARP expression in patient-derived GB cell lines. (**a**) Confocal images show a strong GARP expression on the surface, intracellular (IC) and intranuclear (IN) in all tested patient-derived GB cell lines. The white bar corresponds to 20 µm (**b**) Flow cytometric analysis of the surface expression of GARP. All three cell lines showed an expression of GARP. Due to the nature of these cells, Nestin instead of DiO was stained. Means were normalized to the unstained control.

In order to confirm the expression of GARP on GB tumor cells, a commercially available GB cell line (T98G), three patient-derived GB cell lines (#1043, #1051, #1063), and a melanoma cell line (MaMel-19) were analyzed by flow cytometry and confocal microscopy (Figure 3 and Figure 4). As a positive control for GARP expression, resting and activated Treg were investigated (Figure 3). Flow cytometry data showed GARP localization on the surface of Treg and all tested cell lines, confirming not only previous results but also the in situ data from primary brain tumor tissue (shown in Figure 2) [19].

Interestingly, while analyzing the expression of GARP in brain tumor cells in more detail, we detected intracellular (IC) and intranuclear (IN) localization of GARP in T98G, MaMel-19, and all three patient-derived cell lines (#1043, #1051, and #1063), as well as in resting and activated Treg. All cell lines showed a significant expression of GARP in the cytoplasma as well as in the nucleus of tumor cells. The intracellular expression of GARP was even more pronounced when compared to surface expression. This could be shown using confocal microscopy (Figure 3a and Figure 4a) and for the Treg, T98G, and MaMel-19 also via flow cytometry (Figure 3b).

Thus, our data show, for the first time, intracellular GARP expression in tumor cell lines of GB and melanoma, as well as in Treg.

### 2.3. GB Cell Line T98G Suppresses T Effector Cell Function

It is known that the tumor microenvironment (TME) promotes immune escape mechanisms through inhibitory cell populations such as Treg, myeloid-derived suppressor cells (MDSC), and tolerogenic dendritic cells (tolDC), as well as inhibitory factors produced by the tumor cells themselves [23,24,25]. Furthermore, soluble factors secreted by Treg and tolDC, such as IL-10 and TGF-β, promote the immunosuppressive TME which prevents the rejection of the tumor by the immune system and results in tumor expansion and metastasis [26].

**Figure 5:**
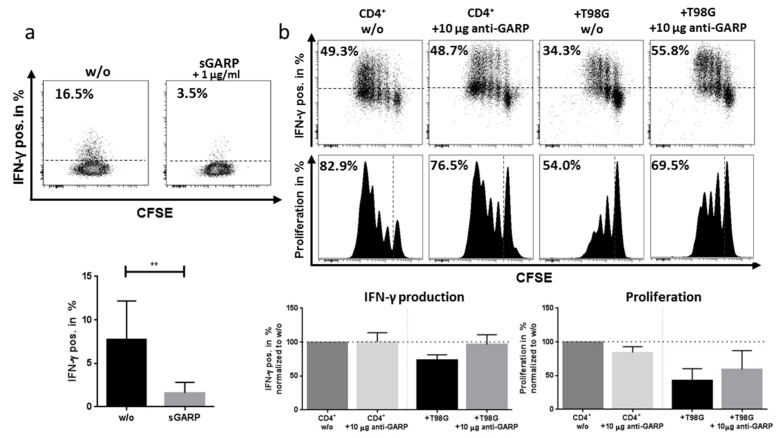
(**a**) Soluble GARP (sGARP) cytokine suppression. CD4^+^ T cells were stimulated with 1 µg/mL anti-CD3 mAb, 1 µg/mL anti-CD28 mAb, and with or without 1 µg/mL sGARP for 24 h. INF-γ production was measured by intracellular staining via flow cytometry. Dot plots show one representative result of 5 independent experiments. (**b**) T98G cells suppress T cell proliferation and cytokine production. CD4^+^ T cells were cultured together with or without (w/o) T98G cells in the ratio of 8:1 and stimulated, as described above. Additionally, either 10 µg/mL anti-GARP Ab or no Ab were added into the culture and CD4^+^ T cells were stimulated as described before. IFN-γ production and proliferation (CFSE) were measured 4 days after stimulation by intracellular staining via flow cytometry. Dot plots show one representative result of 4 independent experiments. Data are displayed as mean values ± SEM, *p*-values relative to w/o ** *p* < 0.01. Dotted lines represent either unstained control (a + b IFN-γ), CFSE stained cells before stimulation or percentages normalized to the untreated control (w/o).

In order to analyze the effect of GB cell line T98G on T effector cells, coculture experiments were performed as described by [19,21] and proliferation and cytokine production of T effector cells were analyzed. As shown previously, the addition of soluble GARP (sGARP, 1µg/mL) downregulated IFN-γ production in activated CD4^+^ T effector cells (Figure 5a). Furthermore, we observed that the addition of T98G to CD4^+^ T effector cells exerted a dose-dependent inhibition of INF-γ production (approximately 30% inhibition), which was nearly completely restored by using a blocking anti-GARP Ab. Proliferation of T effector cells was also inhibited in coculture was, in part, rescued (Figure 5b) by blocking GARP. These results are in agreement with data obtained previously, showing the T effector cell suppression by melanoma cells [21].

Taken together, our data show that GARP plays an important role in the suppressive capacities of GB on T effector cells.

## 3. Discussion

A protein specifically expressed by activated Treg is the activation marker GARP. We have recently shown that GARP (glycoprotein A repetition predominant) has tolerance-inducing functions (inhibition of effector cell proliferation and cytokine production, induction of Treg, and induction of M2 macrophages) [18].

GARP is required for the formation and surface expression of latent TGF-β [19,27] known to be involved in several immunoregulatory mechanisms, especially in tumor biology. The lentiviral knockdown of GARP in Treg showed decreased suppressive capacity and reduced FoxP3 expression in these cells [20]. In addition to its expression on activated Treg, we also showed an occurrence on cells of the malignant melanoma, and thus a further regulatory effect in the tumor micromilieu [21].

GARP has been described by several groups as a transmembrane protein whose extracellular portion consists of 21 leucine-rich domains and is expressed on both Treg and platelets [19,20]. Leucine-rich domains (LRRs) have been identified in a variety of proteins involved in many different functions including signal transduction, cell differentiation, and migration. Those proteins are often membrane bound, can also be secreted or exhibit a cytoplasmic or nuclear localization [28], and are involved in protein–protein interactions. Amongst others, LRRs are found in molecules such as adhesion molecules, enzymes, or tyrosine kinase receptors (RTKs). Despite the localization of RTKs at the cell surface, several are also found in the nucleus [29] being responsible for protein–protein interactions. Whether this is transferable for the intranuclear role of GARP in tumor cells as well as in Treg will be analyzed in more detail in future studies. Nevertheless, structural parallels such as LRRs suggest the possibility of comparable functions also for GARP.

Immunotherapy and targeted therapies have become increasingly important for the treatment of malignant tumors in recent years. However, only some patients respond here. Before considering the increasing number of possible therapy options, the side effects, and the response rates, as well as the costs, it is very important to create a treatment concept individually for each patient with the help of biomarkers. There are numerous efforts to identify factors at the cellular level, as well as at the level of soluble proteins, in the tissue and in the peripheral blood of tumor patients, which help to more accurately characterize the tumor microenvironment and thus the prognosis and the therapy response of an individual patient, and additionally lead to the development of new immunotherapeutic approaches [30,31,32]. In this study, regulatory components of the tumor itself play an important role. In melanoma cells, GARP has been shown to be expressed on the surface of tumor cells, modulating and inhibiting antigen-specific T effector cell responses, and inducing peripheral regulatory T cells [21], thus, contributing to the immune-inhibitory tumor microenvironment. In order to analyze the suppressive role of GARP in GB we used a suppressor assay already published for melanoma cells [21]. In this study, tumor cells were cocultured with T effector cells and IFN-γ production and proliferation of T cells were assessed. We have shown that the presence of GARP on GB cells was, in part, responsible for reduced T effector cell function also showing its immunosuppressive role in GB. The presence of GARP on GB cells may, therefore, be of great importance when discussing prognosis and therapeutic approaches in this tumor entity.

Glioblastoma (GB) is the most common and most malignant form of intrinsic brain tumors accounting for 52% of all primary brain and central nervous system (CNS) malignancies in adults. The current standard of care for newly diagnosed GB is based on the “one-treatment-for-all” principle and consists of surgical resection followed by aggressive regimens of combined radiochemotherapy. Despite aggressive treatment, GBs have a final mortality rate close to 100%, less than a 10% five-years survival rate and a median survival of 15 months [5]. The inevitable recurrence after standard therapy poses a major challenge for improving clinical outcomes of patients with GB. For recurrent GBs (recGBs), no effective therapeutic options are currently available with experimental treatments being the only option at this stage of the disease [33,34]. Currently, immunotherapy is considered among the most promising approaches for recurrent GB, particularly, the targeting of inhibitory T cell signaling mediated through programmed death 1 (PD-1), the PD-1 ligand or cytotoxic T-lymphocyte associated antigen 4 (CTLA-4) has emerged as a promising approach [34]. On the basis of our findings, showing GARP being expressed on activated Treg as well as on brain tumors, it may be an interesting target for new immunotherapeutic approaches using antibody-based strategies.

In Treg, low levels of intracellular GARP were demonstrated prior to activation via the T cell receptor and CD28 [35], suggesting that low levels of GARP are sequestered intracellularly and T cell activation is necessary for the synthesis and surface expression of GARP. In addition, previous studies have shown that ectopically expressed GARP in T cells is able to upregulate Foxp3, indicating a more upstream induction of a tolerogenic phenotype [20]. Interestingly, GARP has also been shown, via Northern blot, to be expressed intracellularly in different tissues such as placenta, lung, kidney, heart, liver, skeletal muscle, and pancreas but not in the brain [28].

The intranuclear localization and accumulation of GARP in cancer cells and also in Treg, as shown in our study for the first time, could be a hint for a second, TGF-β pathway independent way to exerpt its tumor immunity suppressing function. For example, RTKs, proteins containing LRRs similar to GARP protein, are mainly localized at the cell surface. Nevertheless, several RTKs, such as colony stimulating factor 1 receptor (CSF-1R), are also found in the nucleus [29] where they interact with transcription factors regulation cell proliferation, survival, and migration. These full-length proteins translocate from the cell surface to the nucleus via the Golgi apparatus and the endoplasmatic reticulum.

Nevertheless, detailed information about a possible dynamic interaction of GARP with other proteins in the nucleus, and thus potentially regulating gene expression is still elusive and will be a topic for further research.

In the present study we describe for the first time the expression of the immunoregulatory molecule GARP in the tumor microenvironment of primary brain tumors such as GB but also astrocytoma grade II or III. Having shown previously the relevance of GARP for immunomodulation and inhibition of tumor-antigen specific effector cells [21] in melanoma patients, these findings could contribute to the understanding of tumor escape mechanisms of GB including progression and therapy resistance. Notably, GARP is known to exert its function in suppressing tumor immunity via the TGF-β pathway [18], which is one of the key pathways involved in GB progression and maintenance of self-renewal in glioma stem cells (GCS) [36]. The necessity of targeting factors that contribute to the tumor immunosuppressive microenvironment has been increasingly recognized as a strategy to improve the efficacy of immunotherapy for GB [37,38]. Further studies with larger groups of patients are needed to confirm these findings.

Taken together, the present study will help to develop new immunotherapeutic approaches targeting GARP on Treg as well as on GB tumor cells as one possible factor to improve the outcome of GB patients. 

## 4. Materials and Methods 

### 4.1. Cell Culture 

For the cell line T98G, Eagles minimum medium supplemented with 10% FCS, 1% glutamine, and 0.1% primocin was used. The MaMel-19 was cultured with RPMI-1640 supplemented with 10% FCS, 1% glutamine, and 0.1% primocin. The human melanoma cell line MaMel-19 was described previously [21]. Cells were detached via Trypsin-EDTA for 5 min every 3 to 4 days. Cell lines were authenticated at Eurofins Genomics (Ebersberg, Germany) in March 2019. The resulting STR profiles were matched with the online databases of the german collection of microorganisms and cell cultures (DSMZ) (Available online: http://www.dsmz.de/de/service/services-human-and-animal-cell) and Cellosaurus database (Available online: https://web.expasy.org/cellosaurus/) references.

Human glioma cell lines #1043, #1051, and #1063 used in this study were derived from glioblastoma previously described by [39,40,41]. The glioma cells were maintained under a serum-free culture condition that supported cell self-renewal and was based on NeuroBasal Medium supplemented with B27 supplement (Invitrogen, Darmstadt, Germany) and recombinant human cytokines basic fibroblast growth factor 2 (bFGF) and epidermal growth factor (EGF), (10 and 20 ng/mL, respectively, Biochrom GmbH, Merck KGaA, Darmstadt, Germany).

### 4.2. Isolation and Stimulation of Human CD4^+^ T Cells and Treg

Buffy coats were obtained from healthy volunteers, with approval by the local ethical committee (Landesärztekammer Rhineland Palatine No. 837.019.10 (7028), approved on 4 March 2010). The CD4^+^ T cells were isolated via CD4 Microbeads (Miltenyi # 130-045-101). The regulatory T cells were isolated with the CD4^+^ CD25^+^ CD127^dim/−^ isolation kit (Miltenyi #130-094-775, Bergisch Gladbach, Germany) according to the manufacturer’s protocol. For proliferation assays, CD4^+^ T cells were labeled with carboxyfluorescein succinimidyl ester (CFSE, eBioscience #65-0850-84, San Diego, CA, USA) and cultured in 48 well plates at 10^6^ cells/mL, stimulated with 1 μg/mL anti-CD3 mAb (clone OKT3) plus 1 μg/mL anti-CD28 mAb (clone 28.2, eBioscience, San Diego, CA, USA) in the presence or absence of T98G in the ratio of 8:1, 10 μg/mL anti-GARP Ab (Origene AP17415PU-N, Rockland, MD, USA) and 1 μg/mL soluble GARP (recombinant human LRRC32/GARP protein #6055-LR-050, Minneapolis, MN, USA). For the activation of Treg, 1 x 10^6^ cells were stimulated with 1 μg/mL anti-CD3 mAb (clone OKT3) plus 1 μg/mL anti-CD28 mAb (clone 28.2, eBioscience, San Diego, CA, USA) with 10 U/mL IL-2 (Novartis #PZN 02238131, Basel, Switzerland) for 48 h.

### 4.3. Flow Cytometry 

For the flow cytometric analysis, the following antibodies were used: FVD506 (eBioscience #65-0866-14) and GARP (Miltenyi #130-103-820). Cells were stained with fixable viability dye prior to the antibody staining of GARP. Flow cytometry was performed on a BD LSRII flow cytometer (Heidelberg, Germany) and was analyzed using Cytobank [42].

For intracellular and intranuclear staining of GARP or intracellular staining of IFN-γ, cells were fixed and permeabilized with either the intracellular staining kit (BD Cytofix/Cytoperm Plus #555028, Heidelberg, Germany) or intranuclear with the Foxp3 / Transcription Factor Staining Buffer Kit (eBioscience #00-5523-00) and subsequently stained with anti-GARP mAb (Miltenyi #130-103-820) or anti-INF-γ (BD Biosciences #557643, Heidelberg, Germany). 

### 4.4. Confocal Microscopy

For the confocal imaging, the Leica SP8 with HyD Detector (Wetzlar, Germany) was used. Melanoma cell line MaMel 19 and GB cell line T98G were cultured for 24 h in ibidi 15 µ-slides (Ibidi-# 80826, Gräfelfing, Germany), 25,000 cells/well each. Treg were plated on microscopyslides 100,000 each, using a Cytospin centrifuge (Cellspin II- Tharmac, Waldolms, Germany). Cells were checked for adherence and then fixed and permeabilized with a Foxp3/Transcription Factor Staining Buffer Kit (eBioscience, San Diego, CA, USA). For analysis of intracellular localization, cells were stained with anti-GARP mAb for 20 min at RT. Additionally DNA (Hoechst 33342 Solution Promokine #PK-CA707-40046, Heidelberg, Germany) and the membrane (NeuroDiO Solution #PK-CA707-30021- PromoKine, Heidelberg, Germany) were stained for 30 min at RT each. 

For confocal imaging of the non-adherent human glioma cell lines #1043, #1051, and #1063, 30,000–50,000 cells were seeded on glass coverslips pre-coated with poly-L-ornithine hydrobromide (15 µg/mL, Sigma Aldrich, St. Louis, MO, USA) and cultured for 24 h. The cells were fixed with 4% paraformaldehyde/PBS (Merck KGaA, Darmstadt, Germany) for 5 min at RT followed by methanol/acetone (50% v/v) fixation at −20 °C. Cell permeabilization was performed using 0.3% Triton X-100/PBS (Sigma, St. Louis, MO, USA) for 5 min at RT. The primary antibodies used in the study included α-nestin (Abcam ab22035, Cambridge, UK), α-GARP (Origene AP17415PU-N, Rockland, MD, USA), and secondary antibodies (goat α-mouse Alexa Fluor 488 or goat α-rabbit Alexa Fluor 555, Thermo Fisher Scientific, Waltham, MA, USA). Because of their different nature as compared with the adherent T98G, MaMel-19, and non-adherent Treg, α-nestin was used instead of NeuroDiO Solution (see above). For editing, ImageJ2 (Available online: https://imagej.net/ImageJ2) was used [43].

### 4.5. GARP-Immunohistochemistry

Paraffin-embedded tumor samples were studied from 37 GBs (WHO grade IV), 13 anaplastic astrocytomas (WHO grade III), and 6 low-grade astrocytomas (WHO grade II) by GARP immunohistochemistry. Tumor tissue was resected in the Department of Neurosurgery in Idar-Oberstein, Germany, and completely sent for neuropathological examination to the Institute of Neuropathology, University Medical Center Mainz, Germany. Tissue not used for diagnostic proposes was used for additional GARP staining. Written informed consent of all patients was obtained for “scientific use of tumor tissue not needed for histopathological diagnosis” in the admission contract of Idar-Oberstein hospital. Immunohistochemistry was performed on 4 µm thick routinely processed formalin-fixed and paraffin-embedded tissue sections. After dewaxing, antigen retrieval using EnVision FLEX Target Retrieval Solution (), high pH (Dako #S2368 Glostrup, Denmark) was performed. Afterwards, endogenous peroxidase was blocked by peroxidase blocking solution (DAKO, Glostrup, Denmark) and sections were stained with anti-GARP primary antibody 1:100 (Origene AP17415PU-N, Rockland, MD, USA) using an immunostainer (Dako Autostainer Plus, DAKO, Glostrup, Denmark). Immunoreactivity was visualized by the universal immuno-enzyme polymer method (Nichirei Biosciences, Tokyo, Japan). Finally, sections were developed in diaminobenzidine (Lab Vision Cooperation, Fermont, CA, USA). Omission of the primary antisera in a subset of control slides resulted in no immunostaining at all. Nuclear GARP-immunostaining was semiquantitatively assessed in areas with labeled nuclei of tumor cells (more than 90%, 50%, and 10%). Immunohistochemical analysis was performed by an experienced neuropathologist (CS).

### 4.6. Statistics

Results represent the mean ± standard error of the mean (SEM). Statistical significance was determined using the Student’s *t*-test with * *p* < 0.05, ** *p* < 0.01, *** *p* < 0.001 and n.s. (not significant) as indicated.

## 5. Conclusions

Our data indicate for the first time a key role of the immunoregulatory molecule GARP in the tumor microenvironment of primary brain tumors such as GB and low-grade gliomas inducing and promoting tumor immune tolerance via multiple pathways. Moreover, since GARP is not only expressed by activated Treg but also by brain tumor cells, it may serve as a potential target for an immunotherapeutic approach in patients with cerebral cancer.

## Figures and Tables

**Table 1:** Patients with glioma grade III and IV at the study center Idar-Oberstein, Germany were included. Patient characteristics (gender, age), as well as primary tumor data including localization, therapy, and follow-up are displayed.

Glioma Grade III and IV	Male	Female	Total	Lost To Follow Up
Number of patients	26	11	37	
**Age at the onset** **mean±SD (yr.)**	69.05 ± 11.08	71.38 ± 11.72	68.78 ± 13.36	
**Side hemispheric**				
**right**	9	3	12	
**left**	17	8	25	
**bilateral**	0	0	0	
**Surgery**				
**resection**	19	3	27	
**biopsy**	7	8	10	
**First histological diagnosis**				
grade IV	23	10	33	
grade III	3	1	4	
**Localization**				
frontal	5	5	10	
parietal	6	2	8	
temporal	9	2	11	
thalamic	2	0	2	
fronto-parietal	0	1	1	
occipital	2	0	2	
perieto-occipital	0	1	1	
temporo-parietal	2	0	2	
**Radiation therapy**	20	9	29	3
**Chemotherapy**	20	6	26	5
**Survival** **mean ± SD (mon.)**	11.68 ± 15.22	9.38 ± 4.98	11.07 ± 13.27	

## Supplementary Materials

Supplementary materials are found at https://www.mdpi.com/1422-0067/20/15/3676/s1.

## Abbreviations

Treg	Regulatory T cells
GARP	Glycoprotein A repetition domain
GB	Glioblastoma
IC	Intracellular
IN	Intranuclear
sGARP	Soluble GARP
tolDC	Tolerogenic dendritic cells
MDSC	Myeloid-derived suppressor cells
GSC	Glioma stem cells
TME	Tumor microenvironment
LRR	Leucine-rich domains
RTK	Tyrosine kinase receptors
PD-1	Programmed death 1
CTLA-4	Cytotoxic T-lymphocyte associated antigen 4
CFSE	Carboxyfluorescein succinimidyl ester
