# Supplementary material for: GARP as an Immune Regulatory Molecule in the Tumor Microenvironment of Glioblastoma Multiforme
Source: Int J Mol Sci. 2019 Jul 26;20(15):3676. doi: 10.3390/ijms20153676 (PMC6695992; doi:10.3390/ijms20153676)
Supplement: Supplementary file 1 [file ijms-20-03676-s001.pdf]

Table S1. Characteristics of astrocytoma grade II patients at the study center Idar-Oberstein, Germany. Patient characteristics (gender, age), primary tumor data including localization, therapy, and follow-up are displayed.

Low grade Astrocytomas II	male	female	total	lost to follow up
Number of patients	3	3	6	
**Age at the onset mean±SD (yr.)**	60±17.7	55.66±7.6	57.83±12.4	
**Side hemispheric**				
**right**	3	1	4	
**left**	0	2	2	
**bilateral**	0	0	0	
**Surgery**				
**resection**	1	2	3	
**biopsy**	2	1	3	
**Localization**				
midbrain	1	0	1	
Front/temp/insular	1	0	1	
hemispheric	1	0	1	
temporal	0	2	2	
frontal	0	1	1	
**Radiation therapy**	1	0	1	1
**Chemotherapy**	1	0	1	1
**Survival mean±SD (mon.)**	14±15.55	13.5±14.84	13.75±12.41	3

Table S2. Characteristics of astrocytoma grade III patients at the study center Idar-Oberstein, Germany. Patient characteristics (gender, age), primary tumor data including localization, therapy, and follow-up are displayed.

Low grade Astrocytomas III	male	female	total	lost to follow up
Number of patients	6	5	11	
**Age at the onset mean±SD (yr.)**	53.33±13.27	64.2±17.94	58.27±15.7	
**Side hemispheric**				
**right**	3	3	6	
**left**	3	0	3	
**bilateral**	0	2	2	
**Surgery**				
**resection**	4	1	5	
**biopsy**	2	4	6	
**Localization**				
frontal	3	1	4	
temporal	3	1	4	
bifrontal	0	2	2	
hemispheric	0	1	1	
**Radiation therapy**	5	3	8	2
**Chemotherapy**	4	2	6	1

**Survival mean±SD (mon.)**	17.8±12.43	5.0	15.66±12.29	4 female
